# Physical activity surveillance in the European Union: reliability and validity of the European Health Interview Survey-Physical Activity Questionnaire (EHIS-PAQ)

**DOI:** 10.1186/s12966-016-0386-6

**Published:** 2016-05-23

**Authors:** Sebastian E. Baumeister, Cristian Ricci, Simone Kohler, Beate Fischer, Christine Töpfer, Jonas D. Finger, Michael F. Leitzmann

**Affiliations:** Department of Epidemiology and Preventive Medicine, University of Regensburg, Regensburg, Germany; Institute for Community Medicine, University Medicine Greifswald, Greifswald, Germany; Department of Epidemiology and Health Monitoring, Robert Koch Institute, Berlin, Germany

**Keywords:** Validation, Physical activity questionnaire, Recommendations, Population surveillance

## Abstract

**Background:**

The current study examined the reliability and validity of the European Health Interview Survey-Physical Activity Questionnaire (EHIS-PAQ), a novel questionnaire for the surveillance of physical activity (PA) during work, transportation, leisure time, sports, health-enhancing and muscle-strengthening activities over a typical week.

**Methods:**

Reliability was assessed by administering the 8-item questionnaire twice to a population-based sample of 123 participants aged 15-79 years at a 30-day interval. Concurrent (inter-method) validity was examined in 140 participants by comparisons with self-report (International Physical Activity Questionnaire-Long Form (IPAQ-LF), 7-day Physical Activity Record (PAR), and objective criterion measures (GT3X+ accelerometer, physical work capacity at 75 % (PWC_75%_) from submaximal cycle ergometer test, hand grip strength).

**Results:**

The EHIS-PAQ showed acceptable reliability, with a median intraclass correlation coefficient across PA domains of 0.55 (range 0.43–0.73). Compared to the GT3X+ (counts/minutes/day), the EHIS-PAQ underestimated moderate-to-vigorous PA (median difference -11.7, *p*-value = 0.054). Spearman correlation coefficients (ρ) for validity were moderate-to-strong (ρ’s > 0.41) for work-related PA (IPAQ = 0.64, GT3X + =0.43, grip strength = 0.48), transportation-related PA (IPAQ = 0.62, GT3X + =0.43), walking (IPAQ = 0.58), and health-enhancing PA (IPAQ = 0.58, PAR = 0.64, GT3X + =0.44, PWC_75%_ = 0.48), and fair-to-poor (ρ’s < 0.41) for moderate-to-vigorous aerobic recreational and muscle-strengthening PA.

**Conclusions:**

The EHIS-PAQ showed good evidence for reliability and validity for the measurement of PA levels at work, during transportation and health-enhancing PA.

**Electronic supplementary material:**

The online version of this article (doi:10.1186/s12966-016-0386-6) contains supplementary material, which is available to authorized users.

## Background

Insufficient physical activity (PA) is the fourth-leading risk factor for premature mortality in western Europe and among the top 10 globally, causing about 5.3 million deaths per year [1–3]. Physical inactivity has increased substantially during the past decades in the Western world; about one third of adults worldwide do not engage in physical activity at sufficient levels [2, 4].

As the evidence of the adverse effects of physical inactivity accumulates, international policy frameworks have begun to acknowledge the importance of PA [5, 6]. Policy development and evaluation depend on consistent, understandable assessments of prevalence and trends in physical activity and adherence to PA recommendations. Continued improvements in monitoring PA are needed to guide development of policies and programs to increase activity levels and to reduce the burden of chronic disease. Consequently, the WHO and the European Commission have published policy guidelines to provide support in developing related policies [7, 8].

Recently, a health-enhancing physical activity (HEPA) policy audit tool for successful implementation of a population-wide approach to PA promotion across the life course was released by the WHO Regional Office for Europe [9]. A key element of the HEPA tool is the development and implementation of national PA guidelines [9]. So far, less than 40 % of all 53 countries of the WHO European Region have developed national PA recommendations [10]. The European Health Interview Survey (EHIS) is an integral part of the European Commission’s European Core Health Indicators [11], a component of the EU public health surveillance system, which provides data regarding PA prevalence and trends for cross-country comparisons.

Between 2006 and 2010, population-based data for the first cycle of the EHIS were collected in member states of the European Union. PA was assessed using a modified version of the International Physical Activity Questionnaire – Short Form (IPAQ-SF) [12–15]. The IPAQ-SF was developed to facilitate surveillance based on a global standard and is one of the most widely used PA questionnaires [12, 15]. However, although the psychometric properties of the IPAQ-SF have been established and proven to be acceptable [12, 14, 15], the first EHIS wave revealed major difficulties with the IPAQ-SF during data collection [13]. Expert focus groups and cognitive testing studies showed substantial problems with regards to understanding different PA intensity levels, to indicate durations of routine activities such as walking or sitting, and to combine multiple activities to provide the total amount of PA [13, 16]. Consequently, the EHIS Core Group (a group of national health survey experts) commissioned development of the European Health Interview Survey-Physical Activity Questionnaire (EHIS-PAQ), a short, domain-specific PA questionnaire, which allows for the estimation of indicators for total PA, work-related PA, transportation-related PA, and health-enhancing leisure-time PA [13].

The current study examined the reliability and validity of the EHIS-PAQ in a population-based sample of adults drawn from a city in southern Germany. First, 30-day test-retest reliability was examined. Second, to assess concurrent (inter-method) validity, the correlations of the EHIS-PAQ with the International Physical Activity Questionnaire-Long Form (IPAQ-LF), a 7-day PA record (PAR), accelerometer, cardiorespiratory fitness, subjective performance limits, and hand grip strength were tested.

## Methods

### Study sample

A priori sample size calculations for statistical indices of test-retest reliability indicated that about 150 participants would be required to achieve sufficient statistical power [17]. Based on published trends of participation rates of population surveys, we anticipated that approximately 20 % of sampled individuals would agree to participate in the examination [18, 19]. Thus, we sampled 740 individuals aged 18 to 79 years (stratified by age and sex) from the population registry of the city of Regensburg in southern Germany and invited them to participate in the study. Baseline examinations were performed between November 25, 2013 and February 14, 2014 at the University of Regensburg among 140 of the 740 invited individuals (response proportion = 18.9 %). Approximately 30 days after the baseline examination, study participants were sent a resurvey questionnaire and 138 individuals (98.6 %) responded. Complete data were available from 123 participants for the reliability study and, depending on the number of missing values, between 75 and 140 for the validity study.

### Measurements

Data were collected by certified personnel with respect to participants’ socio-demographic characteristics, anthropometry, PA, cardiorespiratory fitness, and hand grip strength. Anthropometric measurements were conducted with participants in light underwear and not wearing shoes and they included height, weight, and hip and waist circumferences. Height was measured in centimeters using a digital stadiometer (SECA Stadiometer 274, SECA, Hamburg, Germany) and weight was measured in kilograms using a digital scale (SECA mBCA 515, SECA, Hamburg, Germany). The body mass index (BMI) was computed using the ratio of weight and height in meters squared (i.e., kg/m^2^).

### EHIS-PAQ

The EHIS-PAQ consists of 8 items covering physical activities during work, transportation, and leisure time (including sports activities), aerobic health-enhancing and muscle-strengthening PA during a typical week [13]. Details regarding the development of the EHIS-PAQ and questionnaire items are provided in Finger et al. [13]. The work-related PA item was taken from the U.S. Behavioral Risk Factor Surveillance Survey [20] and it asked about light, moderate, and heavy and physically demanding activities. This particular item was selected because of its high reliability and validity [21–23]. Four items focused on commuting and active traveling to get from one place to another and inquired about the number of days per week and the time per day spent walking and cycling. Transportation-related walking and cycling items were adapted from the Global Physical Activity Questionnaire (GPAQ) [24] and the IPAQ-LF [12, 14]. The final section of the EHIS-PAQ asked about sports, fitness, and recreational leisure-time physical activities. Sports, fitness, and recreational physical activities were assessed in line with the aerobic PA definition of the CDC/WHO [25–27] but the distinction between moderate and vigorous intensity PA was removed and instead, the item refers to ‘at least moderate intensity’ PA. Participants were asked about how many days and the total duration during a typical week they spent in leisure time sports or fitness pursuits. The last question queried about the days per week the participants engaged in muscle-strengthening PA. The question was adapted from the U.S. National Health Interview Survey - Adult Core questionnaire [28].

We constructed several indices and binary indicators from EHIS-PAQ responses [13]. The work-related PA index summed dichotomous items for light, moderate, and vigorous activities and ranged from 1 to 3. The binary work-related PA indicator compared individuals who ‘mostly sit or stand’ when working with those who perform mostly tasks of ‘at least moderate physical effort’. At an average pace, walking requires about half the energy expenditure of cycling [29]. Thus, we derived a transportation-related PA index (in metabolic equivalent (MET)-minutes per day) by summing the minutes spent walking and cycling, each weighted with MET intensity values (i.e., 3.3 for walking and 6.0 for cycling), provided by Ainsworth’s PA Compendium [29], as suggested by the IPAQ-LF data processing guidelines [30]. A transportation-related PA indicator was generated by grouping participants who fell into the fifth quintile and those who fell into the lower four quintiles of the transportation activity index [13]. According to the WHO recommendations, adults aged 18 years or older should perform at least 150 min of moderate-intensity aerobic PA and at least two occasions of muscle-strengthening PA per week [27]. For constructing an indicator that estimates compliance with the WHO aerobic PA guidelines based on the EHIS-PAQ, information on transportation-related and leisure-time PA can be combined. An index of HEPA was derived by summing the minutes per day spent walking, cycling and engaging in leisure time moderate-intensity PA, where walking minutes were weighted by 0.5 [13, 29]. A recent study indicated that moderate PA is more strongly correlated with objective measurements (accelerometer and heart rate), when walking is excluded [31]. An alternative HEPA index, which was not considered in the current study, could therefore exclude walking from its computation [13]. We also computed the time spent in moderate-to-vigorous aerobic recreational activities (in minutes per day) and muscle-strengthening activities (in occasions per week). Compliance with muscle-strengthening PA guidelines was present when individuals reported at least two occasions of muscle-strengthening PA per week [13, 29]. An indicator of total PA was defined as sufficient aerobic PA according to the WHO recommendation, and being physically active at work.

### Self-reported and objective criterion measures

The self-administered IPAQ-LF used in the present study was based on a validated and back-translated German-language version [12, 14]. The IPAQ-LF was chosen because it evaluates the time and frequency spent in activity domains similar to the EHIS-PAQ. The IPAQ covers four domains of PA: work-related, transportation, household/gardening, and leisure-time activity. For each of the four domains, the number of days per week and the time spent in both moderate and vigorous activity were recoded. To achieve comparability with the outcome measures derived from the EHIS-PAQ, we computed the minutes per day spent in the four activity domains instead of the number of MET-minutes per week, as suggested by the IPAQ-LF data processing guidelines [30]. Participants completed a 7-day PAR regarding activities during work, transportation, leisure-time, and household [32]. For each day, the record consisted of lines of activities grouped into the categories sleep and rest periods, activities at work, leisure time plus home activities, and sports. Total time (in minutes per day) and time spent in each of the four PA domains (i.e., work, transportation, leisure-time, household) were estimated and used as analysis variables. It has been demonstrated that the 7-day PAR is highly correlated with the doubly-labelled water method of energy expenditure assessment [32].

Accelerometer is an established simple, non-invasive and cost-efficient method for objectively measuring PA in a detailed and objective manner [33–35] and were therefore selected as a reference for the concurrent validity study. Participants were asked to wear a GT3X+ accelerometer (ActiGraph LLC, Pensacola, Florida) on a belt at the natural waistline on the right hip in line with the right axilla during daytime and nighttime for seven consecutive days. Data were processed using standard methods; raw data collected from movement registering on the vertical axis were integrated in 60 s periods (epochs). Non-wear time was defined as an interval of at least 60 consecutive minutes of zero counts, allowing for intervals of 1 to 2 min of relatively low counts (i.e., 1-100 counts). Valid wear days were defined as ≥600 min wear time and participants (*n* = 6) with less than three or more valid wear days were excluded [35]. 113 participants provided data for the criterion validity analyses comparing accelerometer data with EHIS-PAQ variables. A cut-off of 1,952 counts per minutes was used to differentiate sedentary-to-light and moderate-to-vigorous activity [36, 37].

Submaximal incremental exercise testing for estimating cardiorespiratory fitness was performed using a calibrated electromagnetically braked cycle ergometer (Ergosana Sana Bike 350/450, Ergosana, Bitz, Germany). Cardiorespiratory fitness is a measure of the capacity of the cardiovascular system to transport oxygen and the capacity of the muscle to use it [38, 39]. The self-reported performance limit (in METs) was estimated using the Veterans Physical Activity Questionnaire (VSAQ) [40]. The VSAQ and the Physical Activity Readiness Questionnaire (PAR-Q) were used for protocol alignment, assessment of the eligibility of the physical fitness appraisal, and derivation of a fitness-adjusted ergometer starting value [40, 41]. The WHO protocol included the following steps: one minute of rest, cycling at a starting workload determined by the VSAQ, and stepwise increases in workload of 25 W every two minutes until 85 % of the estimated age-specific maximum heart rate (i.e., 208-age*0.7 [42]) was exceeded or the maximum intensity level was reached (i.e., 100-175 W) or study personnel terminated the exercise testing due to chest pain, followed by two minutes of recovery [41, 43, 44]. Participants (*n* = 27) were excluded from ergometry if they had a pacemaker, their body weight was >160 kg, they had an elevated blood pressure (≥180/110 mmHg) or resting heart rate (≥100/min), they exhibited ECG abnormalities, arrhythmia, or showed contraindications based on the PAR-Q. The physical work capacity at 75 % (PWC_75%_) of the age-predicted maximum heart rate was determined according to Finger et al. [41] and was used as a primary measure of cardiorespiratory fitness. And 75 participants provided data for the criterion validity analyses comparing the PWC_75%_ with EHIS-PAQ variables.

Muscular fitness is a component of physical function that consists of muscular strength, endurance and power and was used to assess the validity of the EHIS-PAQ question on resistance activity. Isometric grip strength (in kg) was measured in seated position using a hand dynamometer (Jamar Plus+, Sammons Preston, Rolyon, Bolingbrook, IL). Each participant’s grip strength was measured three times with each hand while sitting in a straight-backed chair, feet flat on the floor, shoulders adducted in neutral position, arms unsupported, elbows flexed at 90°, and forearms in neutral rotation. The maximum value (in kilograms) of the six measurements was used [45].

### Statistical analyses

Data on quantitative characteristics are expressed as median (interquartile range) and data on qualitative characteristics are expressed as percent values. Differences between men and women were tested using Kruskal-Wallis and χ^2^ tests. Test-retest reliability was assessed using two EHIS-PAQ administrations spaced approximately 30 days apart and quantified as intraclass correlation coefficient (ICC) and corresponding 95 % confidence limits, estimated using a mixed model [46]. ICCs of >0.5 and >0.7 were considered acceptable and good, respectively [47]. Spearman’s rank ordered correlation coefficients (ρ) were used to measure concurrent (inter-method) validity and the following benchmarks were used for interpretation: 0–0.20 = poor correlation, 0.21–0.41 = fair, 0.41–0.60 = moderate/acceptable, ≥0.6 = strong [48, 49]. The strength of agreement between methods and systematic mis-measurement was assessed using the Bland-Altman technique, which provides the mean bias and the 95 % limits of agreement (±2 standard deviations (SD) of the difference) and is plotted as the difference between the methods against the mean of the methods for visual inspection of error patterns [50]. Median differences between the EHIS-PAQ and accelerometer data were tested using a median regression model. Effect-measure modification with sex (dichotomous), age (continuous), and BMI (continuous) was tested using multiplicative interaction terms in median regression models. The significance level was set at *p* < 0.05. Analyses were performed using SAS 9.3 (SAS Institute, Cary, North Carolina) and PASS 13 Power Analysis and Sample Size Software (NCSS, LLC. Kysville, Utah).

## Results

### Baseline characteristics of study participants

Socio-demographic, behavioral, anthropometric, and measures of cardiorespiratory and musculoskeletal fitness of the 140 study participants are shown in Table 1. The median age was 55 years, about half of the participants were married, more than two thirds had at least 10 years of schooling, and one third reported a history of cardiovascular disease. Men had higher BMI, PWC_75%_, self-assed performance limits, and handgrip strength than women.

**Table 1:** Participant characteristics at baseline (*n* = 140)

		Total	Men	Women	*p*-value*
	N	Median [IQR] or %	Median [IQR] or %	Median [IQR] or %	
N		140	73	67	
Age (years)	140	54.7 [33.1–69.4]	51.0 [31.6–69.7]	57.6 [38.4–69.0]	0.223
Marital status (% married)	140	55.3	60.3	50.0	0.251
Education (% high school or more)	140	71.6	69.9	73.5	0.639
History of cardiovascular disease (%)	140	32.6	37.0	27.9	0.171
Body mass index (kg/m^2^)	140	25.1 [22.4–27.9]	25.8 [23.4–28.2]	23.4 [21.1–27.9]	0.017
PWC_75%_ (Watts/kg)	75	82.1 [41.2–112.4]	104.7 [63.2–139.8]	59.3 [39.2–95.5]	<0.001
Self-assessed performance limit (MET)	114	10 [8–12]	12 [10–13]	10 [8–11]	<0.001
Handgrip strength (kg)	140	33.2 [26.5–46.6]	43.2 [35.5–50.9]	27.6 [22.3–30.8]	<0.001

*PWC*_*75%*_, physical work capacity at 75 %, *MET* metabolic equivalent, *IQR* interquartile range

*p-value from Kruskal-Wallis test

### Physical activity assessed by the EHIS-PAQ

Table 2 provides information regarding binary PA indicators and PA indices of the EHIS-PAQ. In the total sample, 49 % reported work-related PA, about 24 % performed PA during transportation, 38 % had at least two occasions per week of muscle-strengthening activity, 61 % were compliant with the WHO health-enhancing aerobic PA recommendation, and 79 % reported any PA (i.e., achieved the WHO health-enhancing aerobic recommendation or active at work) (Table 2). Compared to women, men had higher values on the transportation-related PA and health-enhancing PA indices, and longer walking time.

**Table 2:** Physical activity assessed by the European Health Interview Survey Interview Physical Activity Questionnaire (EHIS-PAQ) (*n* = 140)

	Total	Men	Women	
	%	%	%	*p*-value
EHIS-PAQ				
Work-related PA indicator	48.6	34.2	64.2	0.112
Transportation-related PA indicator	23.6	21.9	25.3	0.578
Muscle-strengthening PA indicator	37.9	23.9	38.8	0.428
Compliant with HEPA guideline	60.7	53.4	68.7	0.748
Total physical activity indicator	79.3	65.8	79.0	0.475
	Median [IQR]	Median [IQR]	Median [IQR]	
Work-related PA index (1 to 3)	1.0 [1.0–2.0]	1.0 [1.0–2.0]	2.0 [1.0–2.0]	0.061
Transportation-related PA index (MET-min/d)	274.5 [192.1–439.8]	363.2 [211.5–578.3]	269.3 [128.5–395.1]	0.013
Walking time (min/d)	32.0 [19.5–44.5]	32.0 [19.5–44.5]	19.5 [19.5–44.5]	0.041
Cycling time (min/d)	19.5 [19.5–44.5]	19.5 [19.5–32.0]	19.5 [19.5–44.5]	0.871
Moderate-to-vigorous aerobic recreational activity (min/d)	25.7 [17.1–47.1]	27.9 [17.1–49.3]	25.7 [17.1–39.7]	0.391
Muscle-strengthening activity (times/week)	2.0 [1.5–3.5]	2.5 [2.0–4.0]	2.0 [1–2.5]	0.145
HEPA index (min/d)	352.1 [198.7–530.1]	409.3 [224.2–638.5]	292.1 [136.3–447.4]	0.024

*PA* physical activity. *min/d* minutes per day, *MET* metabolic equivalent, *IQR* interquartile range, *HEPA* health-enhancing aerobic physical activity

### Test-retest reliability

ICCs for the 30-day test-retest reliability of the EHIS-PAQ ranged from 0.43 for the health-enhancing PA index to 0.73 for moderate-to-vigorous aerobic recreational PA (Table 3). The repeatability of the work-related PA index and the transportation-related PA were superior to the recall of walking time, cycling time, and muscle-strengthening activity. A higher reliability was noted among men, participants with BMI ≥ 25 kg/m^2^, and older adults (Additional file 1: Table S1).

**Table 3:** Test-retest reliability of the European Health Interview Survey Interview Physical Activity Questionnaire (EHIS-PAQ) (*n* = 123)

	1^st^ administration	2^nd^ administration after 30 days	
EHIS-PAQ	Median [IQR]	Median [IQR]	ICC (95 % CI)
Work-related PA index (1 to 3)	1.0 [1.0–2.0]	1.0 [1.0–2.0]	0.67 (0.57–0.75)
Transportation-related PA index (MET-min/d)	181.35 [64.35–331.35]	146.9 [64.4–263.9]	0.72 (0.61–0.80)
Walking time (min/d)	44.5 [19.5–44.5]	19.5 [19.5–44.5]	0.51 (0.37–0.65)
Cycling time (min/d)	19.5 [19.5–44.5]	19.5 [19.5–44.5]	0.53 (0.28–0.70)
Moderate-to-vigorous aerobic recreational PA (min/d)	25.7 [17.1–51.4]	24.3 [16.2–42.86]	0.73 (0.61–0.82)
Muscle-strengthening activity (times/week)	2.0 [1.0–3.0]	2.0 [1.0–3.0]	0.55 (0.36–0.71)
HEPA index (min/d)	204.8 [68.3–379.8]	155.8 [68.3–292.3]	0.43 (0.23–0.58)

*PA* physical activity, *min/d* minutes per day, *HEPA* health-enhancing aerobic physical activity, *MET* metabolic equivalent, *IQR* interquartile range, *ICC* intraclass correlation coefficient *CI* Confidence intervals

### Concurrent validity

Concurrent validity correlation coefficients of the EHIS-PAQ indices with the IPAQ-LF, PAR, GT3X+, PWC_75%_, and grip strength are shown in Table 4. Strong correlations of the EHIS-PAQ and IPAQ-LF were apparent in the work and transportation domains, and for the HEPA index. Similarly, correlations were moderate in the work domain of the 7-day PAR and between the HEPA index and 7-day leisure time PA. Correlation coefficients were poor-to-fair for the remaining EHIS-PAQ indices.

**Table 4:** Concurrent validity comparing the European Health Interview Survey Interview Physical Activity Questionnaire (EHIS-PAQ) with the International Physical Activity Questionnaire (IPAQ), 7-day Physical Activity Record (PAR), GT3X+ accelerometer, physical work capacity at 75 % maximum heart rate (PWC_75%_), and grip strength

	EHIS-PAQ						
	Work-related PA index (1 to 3)	Transportation-related PA index (MET-(min/d)	Walking time (min/d)	Cycling time (min/d)	Moderate-to-vigorous aerobic recreational activity (min/d)	Muscle-strengthening activity (times/week)	HEPA index (min/week)
	ρ	ρ	ρ	ρ	ρ	ρ	ρ
IPAQ (*n* = 123)							
PA at work (min/d)	0.64**	−0.01	0.15	0.14	−0.01	0.19	−0.10
PA during transport (min/d)	0.05	0.62**	0.58**	0.46	0.23*	0.22*	0.63**
Leisure-time PA (min/d)	0.15	0.33*	0.23*	0.18	0.45**	0.01	0.58**
Household PA (min/d)	0.41**	0.04	0.23*	0.14	−0.08	0.10	−0.10
Total PA (min/d)	0.42**	0.31*	0.33*	0.27**	0.22*	−0.05	0.34*
7-day PAR (*n* = 122)							
PA at work (min/d)	0.47**	−0.03	−0.22	−0.34**	−0.17	−0.13	0.09
PA during transport (min/d)	0.10	0.07	0.13	0.26*	0.12	0.03	0.21*
Leisure-time PA (min/d)	0.38*	0.39*	0.10	0.16	0.51**	0.08	0.64**
Household PA (min/d)	0.37**	−0.01	0.13	0.26*	−0.03	0.11	−0.21*
Total PA (min/d)	0.22*	−0.07	0.05	0.02	0.02	0.09	−0.17
GT3X+ accelerometer (*n* = 113)							
Total activity (counts/min/d)	0.24*	0.43**	0.30*	0.15	0.36*	0.21*	0.43**
MVPA (min/d)	0.06	0.44**	0.31*	0.12	0.32*	−0.006	0.41**
Light activity (min/d)	0.43**	−0.08	−0.01	−0.14	−0.16	0.05	−0.23*
PWC_75%_ (Watts/kg) (*n* = 75)	0.32**	0.01	0.11	0.16	0.25*	0.25*	0.48**
Grip strength (kg) (*n* = 140)	0.48**	0.12	–	0.15	0.14	0.10	0.14

*PA* physical activity, *min/d* minutes per day, *MET* metabolic equivalent. *ρ* Spearman’s rank order correlation coefficients, *HEPA* health-enhancing aerobic physical activity

*p-value <0.05. **p-value <0.05

**Fig. 1:**
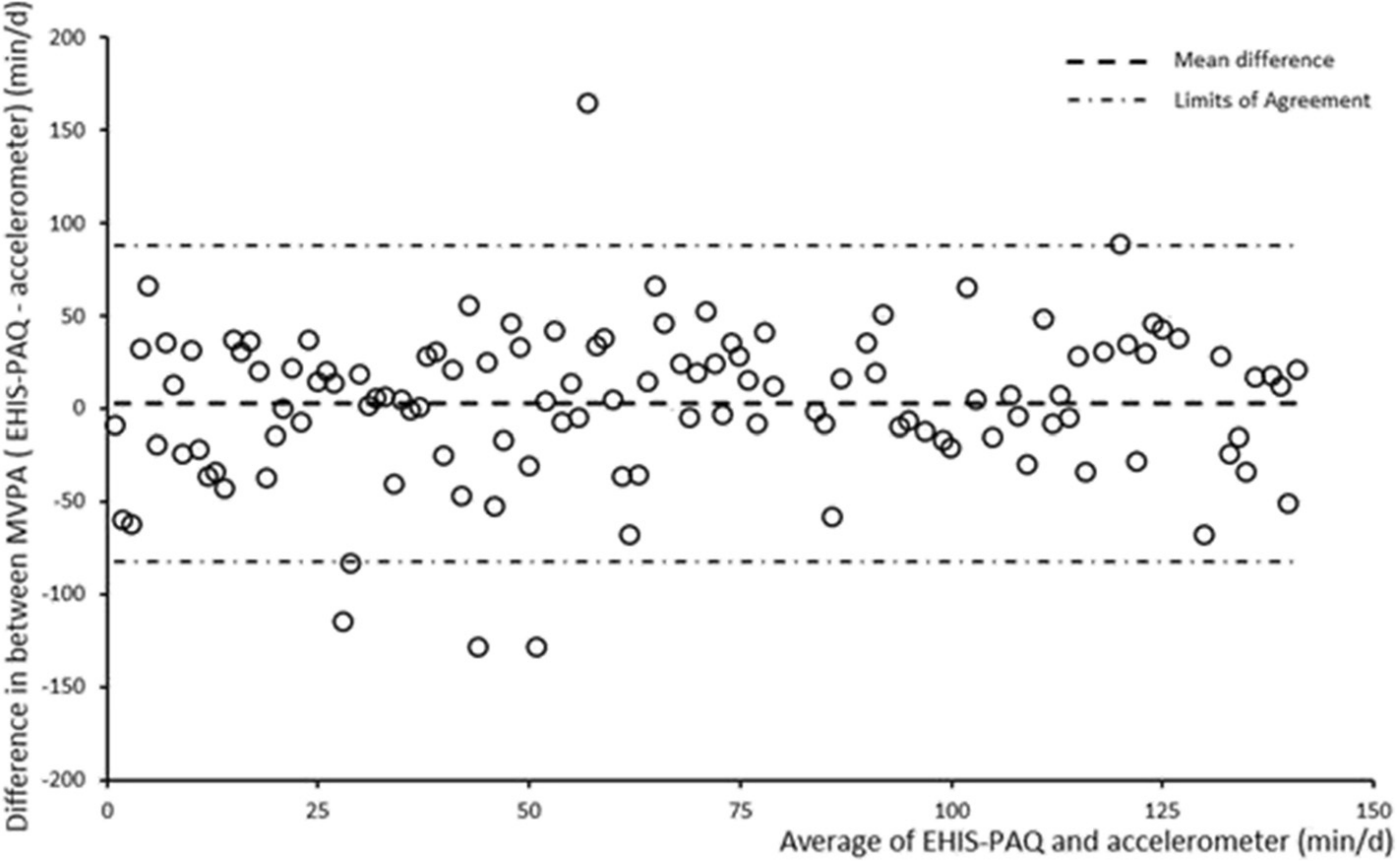
Bland-Altman plot for the agreement of moderate-to-vigorous physical activity (MVPA) from the European Health Interview Survey Interview Physical Activity Questionnaire (EHIS-PAQ) and the GTX3+ accelerometer (*N* = 119). min/d, minutes per day

Moderate correlations with total accelerometer activity were found for transportation-related PA. Correlations between accelerometry-based moderate-to-vigorous activity and EHIS-PAQ domains were moderately strong for transportation activity. Correlations between accelerometry-based light activity and EHIS-PAQ measures were weak, except for work-related PA. Poor-to-fair correlations with accelerometry-based criterion measures were also found for the remaining EHIS-PAQ indices. A fair correlation with PWC_75%_ was noted for the work-related PA and a moderate correlation was seen with HEPA. The association with grip strength was moderate for work-related PA and it was poor to fair for the remaining EHIS-PAQ indices. The median difference between moderate-to-vigorous PA levels from the EHIS-PAQ and the accelerometer was -11.7 min per day; however, the underestimation did not depend on the activity level (Additional file 1: Table S2, Fig. 1). There were no differences in the underestimation of moderate-to-vigorous PA levels by sex, age, and BMI, as indicated by statistically non-significant *p*-values for interaction (Additional file 1: Table S2).

## Discussion

The current study tested the reliability and validity of the EHIS-PAQ, a short PA questionnaire for use in population-based surveillance in a multinational health interview survey context that was developed with the main goals of being easy to complete and enabling estimation of work-related PA, transportation-related PA, leisure PA, HEPA, and muscle-strengthening PA [13]. It was modeled after similar survey items of the IPAQ-LF [12], U.S. Behavioral Risk Factor Surveillance Survey [51], GPAQ [24], and U.S. National Health Interview Survey [28]. The EHIS-PAQ was developed to replace the IPAQ-SF as a PA measurement instrument for the second wave of the EHIS [13]. Field work during data collection of the first EHIS wave, expert groups and cognitive testing had revealed difficulties of study participants answering the IPAQ-SF [13, 16]. Major problems included misunderstanding of the concepts of ‘(light and) moderate’ and ‘vigorous’ PA, classifying activities into different PA intensities, recalling instances of routine activities such as walking, cycling, and sitting, and combining durations of activities in different domains and intensities for calculating the total amount of PA [16]. These problems were particularly evident in respondents aged 60 years or older. Thus, PA surveillance questionnaires used in general population surveys need to consider the cognitive abilities of older people and those with lower reading levels and cognitive abilities [52, 53]. Subsequently, the EHIS-PAQ was developed and pilot tested with the aim of improving understandability, allowing for reliable and valid estimation of PA levels in all population subgroups (including older adults and people with lower cognitive abilities), removing the distinction between intensity levels of PA, defining a minimum intensity level of at least moderate-to-vigorous intensity, and assessing activities of different PA domains [13].

Reliability analyses revealed a median ICC of the EHIS-PAQ of 0.55, with ICCs ranging from 0.43 to 0.73 for individual activity domains. Reproducibility was not modified by gender or age. Taken together, the EHIS-PAQ showed good evidence for reliability, particularity for work-related PA, transportation-related PA, and moderate-to-vigorous aerobic PA. Validity coefficients comparing the EHIS-PAQ questionnaire with self-report and objective criterion measures were stronger and most consistent for HEPA, showing correlation coefficients of 0.58 (IPAQ-LF), 0.64 (7-day PAR), 0.41 (accelerometry), and 0.48 (cardiorespiratory fitness). This highlights the value of the approach taken by the EHIS-PAQ of combining recreational and transportation activity to arrive at an indicator of compliance with the aerobic PA guidelines. By comparison, correlations were slightly weaker for moderate-to-vigorous aerobic recreational activity (disregarding transportation activity), showing correlations between EHIS-PAQ and reference measures of 0.45 (IPAQ), 0.51 (7-day PAR), 0.32 (accelerometry), and 0.25 (cardiorespiratory fitness).

For work-related PA, comparisons of the EHIS-PAQ with the IPAQ-LF and the 7-day PAR yielded moderate/acceptable and strong correlation coefficients of 0.64 and 0.47, respectively. This indicates that distinguishing between individuals who mostly sit or stand and those who perform mostly tasks of at least moderate physical intensity, as is done in the EHIS-PAQ, is particularly useful when assessing occupational activity. For transportation-related PA, we noted a reasonably strong correlation between the EHIS-PAQ and the IPAQ-LF (0.62), which could in part be due to similar question wording and the resulting correlated error structure between those two instruments [12, 14, 15]. By comparison, we found no correlation for transportation activity between the EHIS-PAQ and the 7-day PAR (ρ =−0.07), which may be explained by the fact that the 7-day PAR inquired about work-related transportation activity only and did not assess transportation in other activity domains [32]. Correlation of the EHIS-PAQ with objective criterion measures from the accelerometer, cardio-respiratory exercise testing, and hand grip strength were weaker. For example, the correlation between muscle-strengthening activity from the EHIS-PAQ and hand grip strength was only 0.10 in the overall sample. However, a more pronounced correlation coefficient of 0.42 was found in participants aged 50 years or older. Also, in those aged 50 years or older, muscle-strengthening activity was more strongly associated with cardiorespiratory fitness (0.63) than with grip strength. This suggests that muscle-strengthening activity represents a marker of fitness in middle-aged and elderly individuals and emphasizes the utility of considering population sub-groups in the current validation study.

Our results show that the EHIS-PAQ exhibited psychometric properties that are comparable to established self-report PA questionnaires. Three recent reviews summarized the reliability and criterion validity of common self-report PA questionnaires for adults [15, 53, 54]. According to Helmerhorst et al. [53], few PA questionnaires scored high on both reproducibility and validity, with median reproducibility coefficients of 0.62 to 0.71, and median validity coefficients of 0.30 to 0.41. The IPAQ-SF showed strong repeatability in adults (ρ = 0.76) [12, 53]. A meta-analysis of the validity of the IPAQ-SF reported modest validity correlation coefficients of the total PA level with objective standards in the range 0.09-0.39; none reached the minimal acceptable standard in the literature (i.e., 0.50 for objective activity measuring device; 0.40 for fitness measures) [15, 53, 55]. Time spent walking from the IPAQ-SF showed the highest validity with counts obtained from objective devices, while moderate or vigorous PA correlated weakly with objective standards [15].

This study has several strengths and limitations that need to be considered when interpreting the findings. Participants were recruited using methodologically rigorous general population sampling methods, which helped improve the generalizability of our results. We used objective instruments as reference measures, including an accelerometer, cardiorespiratory fitness, and handgrip strength, thereby enhancing the criterion validity of the study [55]. One methodological limitation is that accelerometers inadequately measure cycling, which was a common activity in our study and may help explain the weak correlation between cycling for transportation from the EHIS-PAQ and moderate-to-vigorous activity based on accelerometry data [56]. In addition, we employed the IPAQ-LF [12] and a 7-day PAR [32] as reference instruments, which allowed us to evaluate the validity of comparable domains of PA. However, recall periods of the self-report questionnaires differ. While the EHIS-PAQ assesses PA during a ‘typical week’, the IPAQ-LF and the 7-day PAR ask about the time participants spent being physically active in the last 7 days. The discrepancy in different time frames can impede comparability because physical activity during the last 7 days might not necessarily reflect the physical activity undertaken in a typical week. For example, physical activity levels are subject to seasonal variation [57] or might be lower when participants are suffering from an acute illness. The modest correlations observed between certain EHIS-PAQ scores and corresponding activity domains based on reference measures indicate that more suitable external criterion measures would have better reflected the EHIS-PAQ scores.

## Conclusions

Overall, the current study examined the reproducibility and validity of the newly developed EHIS-PAQ to monitor PA levels. Findings indicated acceptable-to-good reliability and validity of the questionnaire, which is in good agreement with published review articles and meta-analyses [15, 53, 54]. Notwithstanding some degree of measurement error associated with the EHIS-PAQ, we conclude that the questionnaire quantifies PA and its sub-domains with sufficient validity for use in surveillance studies to inform public policy. Future validation studies should consider using doubly-labeled water as a criterion that despite its high cost remains the recommended standard [15]. Another remaining challenge is to derive harmonized PA measures from the IPAQ-SF and EHIS-PAQ that will allow investigating PA trends based on EHIS waves one and two [58].

## Additional files

Additional file 1: Table S1. 30 day test-retest reliability of the European Health Interview Survey Interview Physical Activity Questionnaire (EHIS-PAQ) by gender, age, and body mass index (BMI). Table S2 Median difference in moderate to vigorous physical activity between the European Health Interview Survey Interview Physical Activity Questionnaire (EHIS-PAQ) and GT3X+ accelerometer in the total study population and stratified by gender, age, and body mass index (BMI). (DOCX 21 kb)

## Abbreviations

BMI: body mass index
CDC: Center for Disease Control
EHIS: European Health Interview Survey
EHIS-PAQ: European Health Interview Survey-Physical Activity Questionnaire
EU: European Union
GPAQ: Global Physical Activity Questionnaire
HEPA: health-enhancing physical activity
ICC: intraclass correlation coefficient
IPAQ-LF: International Physical Activity Questionnaire-Long Form
IPAQ-SF: International Physical Activity Questionnaire - Short Form
IQR: interquartile range
MET: metabolic equivalent
MVPA: moderate-to-vigorous physical activity
PA: physical activity
PAR: 7-day Physical Activity Record
PWC_75%_: physical work capacity at 75 %
SD: standard deviation
VSAQ: Veterans Physical Activity Questionnaire
WHO: World Health Organization
ρ: Spearman’s rank ordered correlation coefficients
