# Supplementary material for: Physical activity surveillance in the European Union: reliability and validity of the European Health Interview Survey-Physical Activity Questionnaire (EHIS-PAQ)
Source: Int J Behav Nutr Phys Act. 2016 May 23;13:61. doi: 10.1186/s12966-016-0386-6 (PMC4877949; doi:10.1186/s12966-016-0386-6)
Supplement: Additional file 1: Table S1. — 30 day test-retest reliability of the European Health Interview Survey Interview Physical Activity Questionnaire (EHIS-PAQ) by gender, age, and body mass index (BMI). Table S2 Median difference in moderate to vigorous physical activity between the European Health Interview Survey Interview Physical Activity Questionnaire (EHIS-PAQ) and GT3X+ accelerometer in the total study population and stratified by gender, age, and body mass index (BMI). (DOCX 21 kb) [file 12966_2016_386_MOESM1_ESM.docx]

Physical activity surveillance in the European Union: reliability and validity of the European Health Interview Survey-Physical Activity Questionnaire (EHIS-PAQ)

Sebastian E. Baumeister ^1,2*^, Cristian Ricci ^1,3^, Simone Kohler ^1^, Beate Fischer ^1^, Christine Töpfer ^1^, Jonas D. Finger ^4†^, Michael F. Leitzmann ^1†^

Additional file (Electronic supplement)

Table S1 30 day test-retest reliability of the European Health Interview Survey Interview Physical Activity Questionnaire (EHIS-PAQ) by gender, age, and body mass index (BMI)

|  | Men | Women | <50 yrs | ≥50 yrs | BMI<25 | BMI≥25 |
| --- | --- | --- | --- | --- | --- | --- |
|  | ICC | ICC | ICC | ICC | ICC | ICC |
| EHIS-PAQ |  |  |  |  |  |  |
| Work-related PA index (1 to 3) | 0.77 | 0.54 | 0.72 | 0.60 | 0.70 | 0.63 |
| Transportation-related PA index (MET-m/d) | 0.70 | 0.25 | 0.60 | 0.46 | 0.27 | 0.79 |
| Walking time (min/d) | 0.51 | 0.46 | 0.56 | 0.46 | 0.53 | 0.45 |
| Cycling time (min/d) | 0.61 | 0.37 | 0.02 | 0.77 | 0.50 | 0.55 |
| Moderate-to-vigorous aerobic recreational PA (min/d) | 0.72 | 0.68 | 0.80 | 0.60 | 0.79 | 0.61 |
| Muscle-strengthening activity (times/week) | 0.43 | 0.58 | 0.68 | 0.30 | 0.63 | 0.87 |
| HEPA index (min/d) | 0.71 | 0.27 | 0.61 | 0.47 | 0.30 | 0.79 |

PA, physical activity. MET, metabolic equivalent. HEPA, Health-enhancing aerobic physical activity.. ICC, intraclass correlation coefficient.

Table S2 Median difference in moderate to vigorous physical activity between the European Health Interview Survey Interview Physical Activity Questionnaire (EHIS-PAQ) and GT3X+ accelerometer in the total study population and stratified by gender, age, and body mass index (BMI)

|  | MVPA from EHIS-PAQ  (min/d) | MVPA from accelerometer  (min/d) | Median difference  (min/d) | p-value* | p-value for interaction† |
| --- | --- | --- | --- | --- | --- |
|  | Median [IQR] | Median [IQR] |  |  |  |
| Overall | 25.7 (17.4-51.4) | 40.8 (26.9-55.4) | -11.7 (-11.0-28.0) | 0.054 |  |
| Men | 30.0 (17.1-60.0) | 36.6 (23.2-55.0) | -1.53 (-29.3-20.1) | 0.538 | 0.146 |
| Women | 25.7 (17.1-51.4) | 41.6 (29.4-54.6) | -14.6 (-7.4-29.5) | 0.004 |  |
| Age < 50 years | 30.0 (19.3-55.7) | 38.9 (26.6-55.6) | -7.48 (-15.9-29.5) | 0.040 | 0.489 |
| Age ≥ 50 years | 25.7 (17.1-51.4) | 40.8 (26.5-55.6) | -12.4 (-6.7-21.1) | 0.710 |  |
| BMI < 25 kg/m² | 32.1 (17.1-51.4) | 38.8 (25.2-55.5) | -12.1 (-9.5-28.1) | 0.229 | 0.528 |
| BMI ≥ 25 kg/m² | 25.7 (17.1-47.1) | 42.6 (29.4-55.0) | -9.30 (-12.3-27.9) | 0.226 |  |

MVPA. Moderate-to-vigorous aerobic recreational. **^*^** p value from median regression. ^†^ p value for interaction from median regression.
